# Bioinformatics Analysis of the Genome of *Rhodococcus rhodochrous* IEGM 1362, an (−)-Isopulegol Biotransformer

**DOI:** 10.3390/genes15080992

**Published:** 2024-07-28

**Authors:** Polina Yu. Maltseva, Natalia A. Plotnitskaya, Anastasiia V. Krivoruchko, Aleksey V. Beletskiy, Andrey L. Rakitin, Andrey V. Mardanov, Irina B. Ivshina

**Affiliations:** 1 Institute of Ecology and Genetics of Microorganisms, Perm Federal Research Center of the Ural Branch of the Russian Academy of Sciences, 13 Golev Str., 614081 Perm, Russia; inbox.98@bk.ru (P.Y.M.); luchnikova.n@mail.ru (N.A.P.); nast@iegm.ru (A.V.K.); 2 Department of Microbiology and Immunology, Perm State University, 15 Bukirev Str., 614990 Perm, Russia; 3 Institute of Bioengineering, Research Center of Biotechnology of the Russian Academy of Sciences, 7-1 Prosp. 60-let Oktyabrya, 117312 Moscow, Russia; mortu@ya.ru (A.V.B.); andrey-rakitin@yandex.ru (A.L.R.); mardanov@biengi.ac.ru (A.V.M.)

**Keywords:** *Rhodococcus rhodochrous*, bioinformatics, (–)-isopulegol, bioactive compounds, whole genome sequence, CYP450

## Abstract

A genome of *Rhodococcus rhodochrous* IEGM 1362 was sequenced and annotated. This strain can transform monoterpene alcohol (–)-isopulegol with the formation of two novel pharmacologically promising metabolites. Nine genes encoding cytochrome P450, presumably involved in (–)-isopulegol transformation, were found in the genome of *R. rhodochrous* IEGM 1362. Primers and PCR conditions for their detection were selected. The obtained data can be used for the further investigation of genes encoding enzymes involved in monoterpene biotransformation.

## 1. Introduction

(–)-Isopulegol ((1*R*,3*R*,4*S*)-*p*-menth-8-en-3-ol, C_10_H_18_O, CAS 89-79-2) is a monoterpene alcohol widely found in the essential oils of plants. Using (–)-isopulegol for the synthesis of novel bioactive compounds has a number of advantages, such as its low cost, availability, and variety of properties [1]. However, the biological transformation of this monoterpene is poorly investigated. There are only fragmented data on the enzymatic conversion of (–)-isopulegol by cutinases from *Aspergillus oryzae* and *Humicola insolens* [2] and its whole cell degradation by *Rhodococcus* sp. [3]. *Rhodococcus* actinomycetes are biotechnologically promising microorganisms due to their broad substrate specificity and high catalytic activity even under extreme environmental conditions [4]. Representatives of *R*. *rhodochrous* have a wide range of applications due to their ability to degrade ecotoxic compounds, such as pharmacological pollutants [5], polycyclic aromatic hydrocarbons [6], and pulp and paper industry waste [7]. Furthermore, they are known to be effective catalysts for the synthesis of bioactive compounds [8,9,10,11], as well as being enzyme sources [12]. In our recent study, *R. rhodochrous* IEGM 1362 was shown to biotransform monoterpene alcohol (–)-isopulegol into two new 10-hydroxy and 10-carboxy derivatives [13,14]. Both compounds may have biological functions, such as antitumor, respiratory-stimulating and anticancer capabilities.

The ability of bacterial cells to transform various compounds is due to the action of their enzyme systems. A logical trend in the development of biotechnology is the isolation and purification of bacterial enzymes, as well as their overexpression in model organisms in order to scale the production of target compounds. The use of individual enzymes allows for highly selective reactions with repeated use, reduces sterility requirements, and simplifies the process of isolation and purification of the target product [15]. In this regard, studies on the catalytic activity of *Rhodococcus* spp. in relation to terpene substrates should be accompanied by the study of microbial enzymes involved in transformations, as well as the functional genes encoding them. Several rhodococcal genes and enzymes have been identified that catalyze the transformations of monoterpenoids 1,8-cineole, limonene, carveol, *p*-cymene, and terpineol [16,17,18,19,20]. However, data on bacterial genes and enzymes for the bioconversion of (–)-isopulegol are lacking. Our research focuses on the primary analysis of the whole genome of *R. rhodochrous* IEGM 1362, an (–)-isopulegol biotransformer, to identify putative genes encoding enzymes involved in (–)-isopulegol bioconversion.

## 2. Materials and Methods

### 2.1. Culture

*R. rhodochrous* IEGM 1362 was isolated from the Paltinskoye peat deposit, Perm region, Russia, and deposited in the Regional Specialized Collection of Alkanotrophic Microorganisms (acronym IEGM, WDCM number 768). Along with (–)-isopulegol transformation, the strain also uses *n*-hexadecane as a sole carbon source, degrades dehydroabietic acid, and is resistant to Cr^6+^ (40.0 mM), Mo^6+^, and Zn^2+^ (5.0 mM) (http://www.iegmcol.ru/strains/rhodoc/rhodoch/r_rhod1362.html, accessed on 4 July 2024).

### 2.2. Whole Genome Sequencing

For DNA extraction, the *R*. *rhodochrous* IEGM 1362 cells were grown in LB broth at 28 °C and 160 rpm for 28 h. Genomic DNA was isolated using a DNeasy PowerSoil Pro Kit (Qiagen, Hilden, Germany). The shotgun library was prepared using an NEBNext Ultra II DNA library prep kit (New England Biolabs, Ipswich, MA, USA) and sequenced on an Illumina MiSeq instrument in paired read mode (2 × 300 nt). A total of 506,369 read pairs were generated. Low-quality sequences were trimmed using Sickle v. 1.33 (q = 30). The draft genome was assembled with Flye v. 2.8.1.

### 2.3. Bioinformatics Analysis

The search for functional genes presumably involved in the biotransformation of (–)-isopulegol and rRNA genes was carried out using the online service RAST (Rapid Annotation using Subsystem Technology, https://rast.nmpdr.org/, accessed on 3 June 2024) [21] based on an automatically annotated whole genome of the biotransformer strain.

The 16S rRNA gene of *R. rhodochrous* IEGM 1362 was aligned with 16S rRNA genes of closely related bacterial strains, and a distance tree for aligned nucleotide sequences was built in BLAST (https://blast.ncbi.nlm.nih.gov/Blast.cgi, accessed on 14 June 2024). Digital DNA–DNA hybridization (dDDH) with the genomes of type strains and phylogenetic analysis were performed on TYGS (the Type (Strain) Genome Server, https://tygs.dsmz.de/, accessed on 14 June 2024) [22]. Average nucleotide identities (ANIs) were calculated using an ANI Calculator (https://www.ezbiocloud.net/tools/ani, accessed on 14 June 2024) [23].

A comparison of the target gene sequences was carried out using the BLASTN and BLASTP services available on the NCBI website. The search for biosynthetic gene clusters in the genome of the biotransformer strain was carried out using genomic mining on the online service AntiSMASH (https://antismash.secondarymetabolites.org/, accessed on 3 June 2024) [24]. An analysis of the amino acid sequences and the construction of metabolic pathways were carried out using the KEGG database (Kyoto Encyclopedia of Genes and Genomes, https://www.genome.jp/kegg/, accessed on 3 June 2024) and the GhostKOALA service [25].

### 2.4. Primer Design

Primers were designed using Primer-BLAST. When selecting them, we were guided by recommendations from the NCBI, the uniqueness of specific regions in the genome, a product length of 70−1000 bp, a melting temperature of 57−63 °C, and a GC composition of 40−60%. In this case, the difference in the GC composition should be no more than 10%, the difference in the melting temperatures between the forward and reverse primers should be no more than 0.99 °C, the self-complementarity indicator should be no more than 8 arbitrary units, and the maximum complementarity of pairs should be no more than 5 arbitrary units.

### 2.5. Determination of Genes Encoding Enzymes Involved in (–)-Isopulegol Transformation

DNA extraction was carried out using biomass obtained after a 24 h cultivation of the strain in an LB nutrient broth (Diaem, Moscow, Russia) according to the protocol for the ExtractDNA Blood genomic DNA isolation kit (Evrogen, Moscow, Russia). The concentration and purity of the isolated DNA were assessed using a Qubit^TM^ fluorimeter (Thermo Fisher Scientific, Waltham, MA, USA) with a QuDye dsDNA BR kit (Lumiprobe, Moscow, Russia) and a NanoPhotometer N50 spectrophotometer (Implen, Munich, Germany), respectively. The resulting DNA was used for PCR with a qPCRmix-HS SYBR (Evrogen, Moscow, Russia) with the selected primers on a CFX Connect^TM^ Real-time system (Bio-Rad, Hercules, CA, USA). Species-specific primers based on the 16S rRNA gene for *R*. *rhodochrous* served as a positive control. The PCR protocol included the following steps and conditions:
Stage 1:95.0 °C; 3 min.Stage 2:95.0 °C; 30 s.Stage 3:Gradient 55.0–65.0 °C; 30 s.Stage 4:72.0 °C; 1:30 min.Repetition of Stages 2–4 34 times.Stage 5:Melting curve from 65.0 to 95.0 °C, step 0.5 °C; 5 s.Stage 6:72.0 °C; 10 min.

The presence and size of amplicons in the reaction mixture after PCR were determined using horizontal electrophoresis in an agarose gel (1.5% agarose in TBE buffer) using a Bio-Rad Gel Doc XR+ gel documentation system (Bio-Rad, Hercules, CA, USA). Electrophoretic separation was carried out at a voltage of 70 V for 40 min. GelRed (Diaem, Moscow, Russia) was used as a nucleic acid dye. PCR products (5 μL) were added to an agarose gel in 4X Gel Loading Dye, Blue loading buffer (0.5 μL) (Evrogen, Moscow, Russia). To determine the size of the PCR products, a DNA length marker from 700 to 50 bp (Evrogen, Moscow, Russia) was added to the gel.

## 3. Results and Discussion

### 3.1. Phylogeny and Overall Genome Characteristics

The assembly consisted of 140 contigs with a total sequence length of 5,733,046 bp, an N50 value of 126,441 bp, a GC content of 68%, and coverage of 53.0×. A total of 5331 CDSs, 5209 CDSs with proteins, and 67 RNAs were found in the *R*. *rhodochrous* IEGM 1362 genome (Table 1).

**Figure 1:**
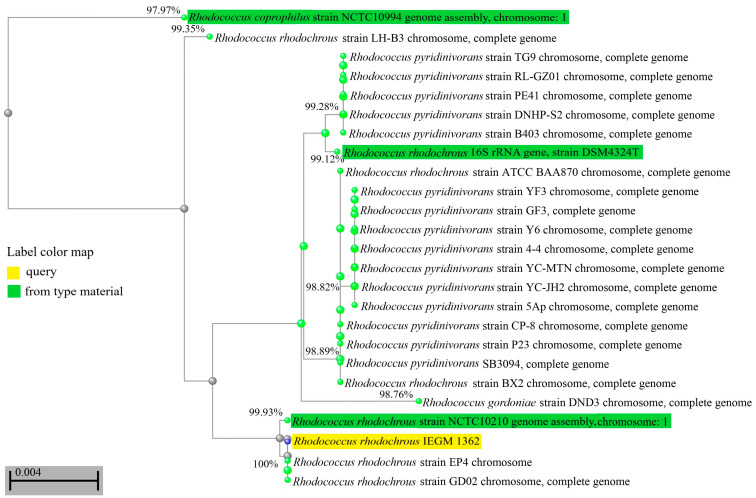
Distance tree for pairwise alignments between 16S rRNA gene sequences of *R. rhodochrous* IEGM 1362 and the most phylogenetically closed strains. The tree method was Fast Minimum Evolution. The tree was unrooted. Only strains with a complete sequence of a 16S rRNA gene were selected for tree production. Values near the edges show the percentages of identity between 16S rRNA genes.

Using phenotypic methods (morphology, physiological tests, chemotaxonomy), the IEGM 1362 strain was identified as belonging to the *R. rhodochrous* species. During the analysis of the genome and phylogenetic markers, such as the 16S rRNA gene, it was revealed that *R. rhodochrous* IEGM 1362 was not a common member of this species. The scores for the key taxonomic markers (16S rRNA gene similarities, dDDH, and ANI) of *R. rhodochrous* IEGM 1362 were close to threshold levels of 98.7%, 70%, and 94–95%, respectively [26]. According to the gene relatedness of 16S rRNA, the *R. rhodochrous* strain IEGM 1362 was in the same phylogenetically close group with the *R. coprophilus* strain NCTC10994, the *R. gordoniae* strain DND3, and various *R. pyridinivorans* and *R. rhodochrous* strains (Figure 1). Only the percent of identity with the 16S rRNA gene of *R. coprophilus* NCTC10994 was below the threshold of <98.7% [26], while the levels of identity with 16S rRNA genes of other strains were ≥98.7% (Figure 1). This indicated that *R. rhodochrous* IEGM 1362 could be classified as *R. gordoniae*, *R. pyridinivorans*, or *R. rhodochrous*.

The dDDH scores were more than 70% in comparisons of the IEGM 1362 genome with genomes of *R. rhodochrous* type strains and less than 70% in comparisons with genomes of *R. gordoniae* and *R. pyridinivorans* type strains (Table 2). This parameter was evidence for the IEGM 1362 strain belonging to *R. rhodochrous*, which agrees with the phenotypic traits of this strain. However, ANI scores were above the threshold of 94–95% for two species, *R. pyridinivorans* and *R. rhodochrous*, and G+C content was less different (0.05–0.25%) between *R. rhodochrous* IEGM 1362 and type strains of *R. gordoniae* and *R. pyridinivorans* than that between the IEGM 1362 strain and type strains of *R. rhodochrous* (0.38–0.45%) (Table 2).

According to the ANI and dDDH values, the IEGM 1362 strain did not belong to *R. gordoniae*. However, its difference from *R. pyridinivorans* was not evident. Summarizing all the scores (the 16S rRNA gene relatedness, the highest ANI score, and dDDH values within the species borders), the IEGM 1362 strain continued to belong to *R. rhodochrous*. Apparently, *R. rhodochrous* IEGM 1362 accumulated genetic traits of both closely related species *R. pyridinivorans* and *R. rhodochrous* and occupied an intermediate position between these two species. The heterogeneity of the *R. rhodochrous* IEGM 1362 genome, distancing this strain from common representatives of *R. rhodochrous*, could be a basis for its advanced catalytic properties, including its ability to transform (–)-isopulegol.

### 3.2. The Whole Genome Analysis

**Figure 2:**
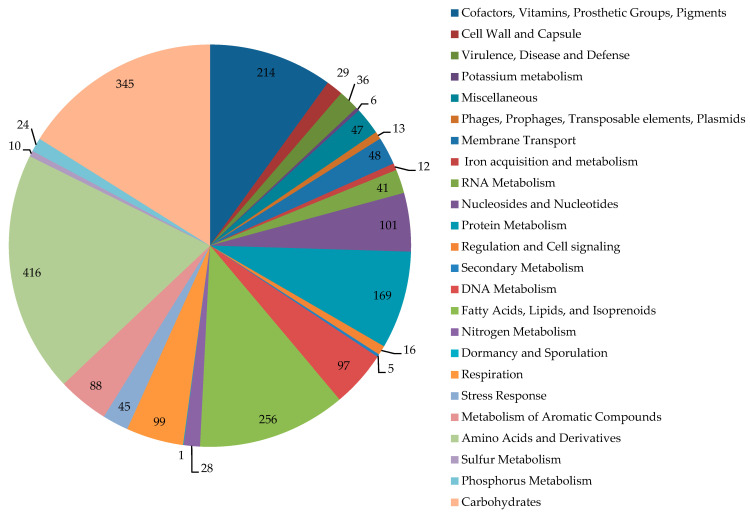
Distribution of subsystem categories in the genome of *R*. *rhodochrous* IEGM 1362. Image obtained using SEED Viewer 2.0.

The category distribution of genes with known functions is shown in Figure 2. In the genome of IEGM 1362, 75 CDSs encoding monooxygenases/hydroxylases, 31 CDSs encoding dioxygenases, 7 CDSs encoding peroxidases, and 351 CDSs encoding dehydrogenases were found. Among monooxygenases, the genes of two alkane 1-monooxygenases, nine cytochrome P450s, eight cyclohexanone 1,2-monooxygenases, three 3-ketosteroid-9-alpha-monooxygenases, and two steroid C27-monooxygenases were revealed. Additionally, we found several genes encoding enzymes of steroid metabolism, such as cholesterol oxidases (six CDSs), 3-oxosteroid 1-dehydrogenases (four CDSs), 3-alpha-hydroxysteroid dehydrogenase (one CDSs), steroid monooxygenases (three CDSs), and 3-ketosteroid-9-alpha-monooxygenases (three CDSs).

Based on the results of the analysis of metabolic pathways in the genome of strain IEGM 1362, a non-mevalonate pathway for C5 isoprenoid biosynthesis was detected, including the following enzymes: 1-deoxy-D-xylulose-5-phosphate synthase (EC 2.2.1.7), 1-deoxy-D-xylulose-5-phosphate reductoisomerase (EC 1.1.1.267), 2-C-methyl-D-erythritol-4-phosphatecytidyl transferase (EC 2.7.7.60), 4-diphosphocytidyl-2-C-methyl-D-erythritol kinase (EC 2.7.1.148), 2-C-methyl-D-erythritol 2,4-cyclodiphosphate synthase (EC 4.6.1.12), 1-hydroxy-2-methyl-2-(E)-butenyl-4-diphosphate synthase (EC 1.17.7.1), and 4-hydroxy-3-methylbut-2-enyldiphosphate reductase (EC 1.17.1.2) (Supplementary Materials, Figure S1) [28]. This pathway provides the formation of isopentenyl diphosphate and dimethylallyl diphosphate, which are necessary for the synthesis of terpenes, sterols, carotenoids, and dolichols.

It should be noted that, at present, the vast majority of processes for the biosynthesis of terpene compounds in actinomycetes have been discovered and studied mainly for representatives of the genus *Streptomyces* [29], whereas for representatives of the genus *Rhodococcus*, only a few examples of the biosynthesis of zeatins, isoprenoid cytokinins with a dimethylallyl moiety attached to the N atom of adenine or adenosine [30,31], and valuable carotenoids such as dihydroxyneurosporine, hydroxyequinenone [32], beta-carotene, zeaxanthin, and isorenieratin [33], etc. [34], have been detected. Since the strain we used in our work is characterized by the red color of the colonies, the discovered pathway is most likely responsible for the synthesis of terpene carotenoids. In addition, using the online genomic mining service AntiSMASH, the presence of three biosynthetic gene clusters for the synthesis of terpenes in the genome, as well as biosynthetic gene clusters for the synthesis of polyketides, non-ribosomal peptide synthases, ectoines, butyro-, and beta-lactones and ε-poly-L-lysine, was shown (Table 3).

### 3.3. Determination of Putative Monoterpenoid Transformation Genes

To date, only a few genes and the enzymes they encode have been identified that catalyze the conversion of individual monoterpenoids [16,17,18,19,20]. Using limonene as an example, the enzymes limonene-1,2-epoxide hydrolase, limonene-1,2-monooxygenase, and limonene-6-monooxygenase, which are responsible for its transformation through hydroxylation, oxidation, and hydrolysis in *R*. *erythropolis* cells, were identified [17]. A gene encoding limonene-1,2-epoxide hydrolase was also found in the genome of IEGM 1362 (fig|6666666.993513.peg.1867), which indicates that this strain may exhibit limonene-transforming activity.

**Figure 3:**
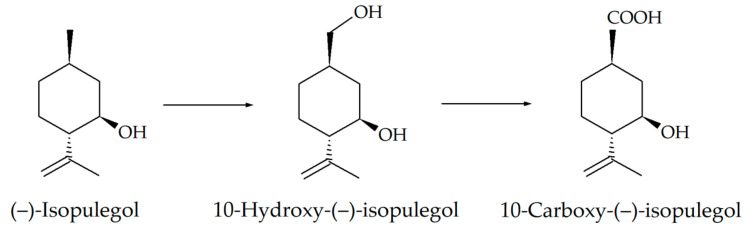
Scheme of (–)-isopulegol biotransformation by *R*. *rhodochrous* IEGM 1362 [13,14].

Based on the recorded transformations of (–)-isopulegol, including the stages of hydroxylation and oxidation (Figure 3), and the analysis of the literature data [35], we assumed that enzymes of the cytochrome P450 family can be involved in the bioconversion process. Automatic annotation of the obtained sequences of *R*. *rhodochrous* IEGM 1362 using the RAST service made it possible to search for genes encoding enzymes presumably involved in the bacterial oxidation of (–)-isopulegol. We discovered nine genes encoding enzymes of the family of cytochrome P450-dependent oxygenases and hydroxylases (Table 4). Pairwise comparison of these genes within strain in the BLAST service showed the absence of significant matches in Megablast mode and no more than 33.5% similarity accordingly to the blastp (protein–protein BLAST) algorithm (Supplementary Materials, Figure S2). This confirms the fact that all of the discovered genes are not copies, but separate functional units. It is worth noting that, based on the results of a search for genes encoding CYP450 in the NCBI database, 13 genes were identified, but the transcription products of 4 of them in the RAST system were identified more specifically as steroid C27-monooxygenases (EC 1.14.13.141) and lanosterol 14-alpha demethylase (EC 1.14.13.70); therefore, they were not used by us in further experiments.

**Figure 4:**
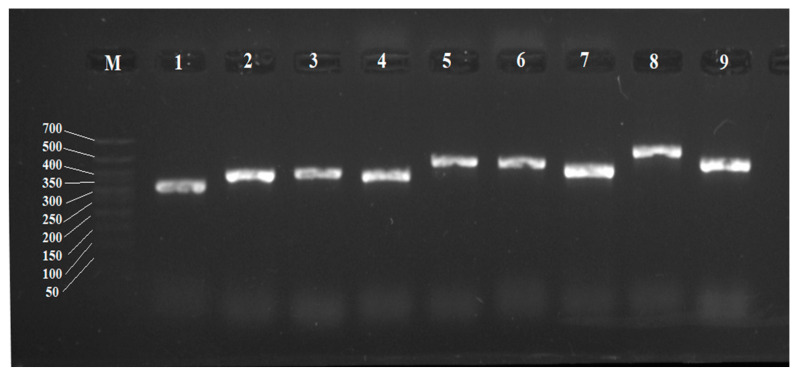
Electropherogram of PCR products of *R*. *rhodochrous* IEGM 1362 with specific primers for CYP450 genes: M, DNA length marker from 700 to 50 bp. The gene numbers are the same as the gene numbers in Table 4.

Based on the detected sequences, for the first time, pairs of primers for individual CYP450 genes of *R*. *rhodochrous* IEGM 1362 were selected using the Primer-BLAST service. Optimal conditions for their amplification were selected. These guaranteed the formation of products with an expected size without the amplification of extra DNA fragments of other sizes typical for GC-rich matrices (Table 4, Figure 4).

Genes encoding CYP450 enzymes in *R. rhodochrous* IEGM 1362 differed from their homologues in other *R. rhodochrous* strains; this could be a basis for the advanced transformation capabilities of this strain. Percents of identity between the CYP450 nucleotide and amino acid sequences varied in wide ranges of 88.72–100.00% and 56.36–100.00%, respectively (Table 5). The highest (100.00%) and the lowest (88.72% and 56.36%) similarities were not typical and were detected in a comparison of *R. rhodochrous* IEGM 1362 with a few *R. rhodochrous* strains. Some of *R. rhodochrous* IEGM 1362 cytochrome genes were rare and unique for bacteria. Gene nos. 4, 6, and 7 (see Table 4) were found only in 13, 59, and 46 bacterial strains. Among *R. rhodochrous* strains, gene no. 6 was unique for the IEGM 1362 strain, since it was not found in other representatives of *R. rhodochrous*. However, according to amino acid sequencing, an enzyme with 94.55% identity with CYP450 no. 6 of IEGM 1362 was detected in one *R. rhodochrous* strain (Table 5). This enzyme was also found in *R. pyridinivorans* with high identities of 99.85–99.92% and 99.77% for DNA and amino acid sequences, respectively (Table 5). The high similarity of *R. rhodochrous* IEGM 1362 cytochromes with those in *R. pyridinivorans* was also evident from the close identities of these enzymes, which varied in ranges of 82.05–100.00% and 56.11–100.00% for DNA and amino acid sequences, respectively (Table 5). Moreover, cytochromes of *R. rhodochrous* IEGM 1362 were more frequently detected in *R. pyridinivorans*. Homologues of these enzymes and their genes were found in 1–27 strains of *R. pyridinivorans* compared with 0–12 strains of *R. rhodochrous* (Table 5). This was not related to the over-representation of sequenced genomes for one of the species in the NCBI. There were 31 strains with sequenced genomes for both species.

The genetic surroundings of CYP450 genes were analyzed. The genes that coded for transcriptional regulators were found directly before or in close proximity to eight CYP450 genes. Only gene no. 6 had no transcription regulators nearby. However, partner proteins for cytochromes, such as ferredoxin and ferredoxin reductase, were detected only in the surrounding of CYP450 no. 6. We supposed that genes encoding CYP450 no. 6, ferredoxin, and ferredoxin reductase consisted of one gene cluster expressed as a polycistron transcript, and that these proteins were responsible for a distinct metabolic process, probably (–)-isopulegol transformation. Other cytochromes in *R. rhodochrous* IEGM 1362 could be involved in complex metabolic processes requiring global regulation along with other genes. Such processes could be the biosynthesis of lipids, fatty acids, or acylated polymers (glycolipids or lipoproteins), since genes coding for corresponding enzymes were found near CYP450 genes. Other metabolic processes could include the neutralization of toxic organic compounds, since genes coding for various transporting proteins and other oxidoreductases were also found near CYP450 genes. Gene no. 8 apparently was involved in the metabolism of aromatic compounds, since genes encoding benzoate transporters, benzoate, and catechol dioxygenases, and some specific dehydrogenases, were annotated in the same gene cluster (Table 6). A common characteristic of cytochromes is their wide substrate specificity. Consequently, any of the CYP450 enzymes found in the genome of *R. rhodochrous* IEGM 1362 can theoretically enable the transformation of (–)-isopulegol, including providing co-oxidation conditions.

Additionally, a perspective of horizontal gene transfer (HGT) of genes coding for cytochrome P450 in *R. rhodochrous* IEGM 1362 was estimated. Mobile elements were only found near three CYP450 genes. Just one mobile element protein was detected in each case (Table 6). Apparently, HGT plays a minor role in the distribution of cytochrome-coding genes between *Rhodococcus* species; however, it is probably responsible for the appearance of the unique CYP450 no. 6 in the genome of *R. rhodochrous* IEGM 1362.

The data obtained expand the understanding of the molecular genetic basis of the transformation of terpenoids by actinomycete cells of the genus *Rhodococcus* and create the prerequisites for further analysis of gene expression levels in order to identify genes and enzymes for the highly efficient selective biotransformation of the plant monoterpenoid (–)-isopulegol.

## Figures and Tables

**Table 1:** Genome features for *R*. *rhodochrous* IEGM 1362 assembly (according to NCBI).

Feature	Value
Size, bp	5,733,046
GC content, %	67.8
N50, bp	126,441
L50	13
Number of contigs	140
Number of coding sequences	5331
Number of RNAs	67
Genome coverage	53.0×

**Table 2:** Overall genomic characteristics of *R. rhodochrous* IEGM 1362 compared with type strains from the TYGS database.

Subject Type Strain	dDDH, % *	G+C Content Difference, %	ANI, %
*R. rhodochrous* NBRC 16069	79.7	0.45	97.86
*R. rhodochrous* NCTC10210	79.4	0.38	97.89
*R. rhodochrous* DSM 43241	79.6	0.45	97.81
*R. pyridinivorans* TG9	60.4	0.25	95.15
*R. pyridinivorans* DSM 44555	58.9	0.05	94.91
*R. gordoniae* DSM 44689	41.6	0.15	90.81

* Formula d4 (GGDC formula 2) was used: sum of all identities found in high-scoring segment pairs (HSPs) divided by overall HSP length [27].

**Table 3:** Biosynthetic gene clusters in the genome of *R. rhodochrous* IEGM 1362.

Coding Element	Number of Clusters
Non-ribosomal peptide synthetase	6
Terpene	3
Ectoine	1
beta-Lactone	2
Butyrolactone	1
ε-Poly-L-lysine	1
T1 Polyketide synthase	1

**Table 4:** Specific primers for genes encoding CYP450 enzymes of *R*. *rhodochrous* IEGM 1362, and optimal PCR conditions for their detection.

Gene No.	Contig ID	Gene Location (Gene Size, bp)	Primer Direction	Primer Sequence (5′ → 3′)	Tm, °C	Amplicon Size, bp
1	NODE_57_length_21710_cov_10.083492	20,649–18,289 (2361)	Forward	TACAGCCCCGAACTCGACTA	55.7	311
			Reverse	TGTCCGGTATCGATGAAGCG		
2	NODE_33_length_49450_cov_11.184397	41,128–42,420 (1293)	Forward	GTTCATCGAGGGCCTGAACA	57	373
			Reverse	CTCGAAACGCAGTGTCTCCT		
3	NODE_17_length_96204_cov_10.945325	67,773–69,119 (1347)	Forward	CGACGACATCTTCTCGGTGT	59	389
			Reverse	TGTGGTGGGAGAAGTGCATC		
4	NODE_54_length_24166_cov_8.603810	6580–7959 (1380)	Forward	TCGTTCGCTTTGCACTACCT	59	370
			Reverse	CGAATGGCTTGTATGCGTGG		
5	NODE_2_length_337154_cov_11.648399	238,563–237,184 (1380)	Forward	CACGTCGACCATCACGATCT	61.4	448
			Reverse	TTGGTGGACGATCGCTTTGA		
6	NODE_27_length_73775_cov_9.719571	62,892–64,214 (1323)	Forward	GCTCTGACGCAGGAGTTCTT	61.4	444
			Reverse	ACGCAGTGTGTAGTCCTGTG		
7	NODE_33_length_49450_cov_11.184397	31,096–29,762 (1335)	Forward	ATCGGTTCACCCAGAACCTG	59	403
			Reverse	GAGGACAGGATCACGAAGCC		
8	NODE_2_length_337154_cov_11.648399	99,355–98,144 (1212)	Forward	CCGATCTCGTAGCCCAGTTC	61.4	554
			Reverse	TTCGCTGATCTCGATTCCCG		
9	NODE_20_length_89047_cov_8.544141	82,615–81,383 (1233)	Forward	CATCTCCCACGGCCTGTATC	63.3	461
			Reverse	TGTACTCGACACGAAGGTGC		

**Table 5:** Percents of identities for genes encoding CYP450 enzymes in *R*. *rhodochrous* IEGM 1362 compared with those in *R. rhodochrous* and *R. pyridinivorans* strains.

Gene No.	Identities for DNA Sequences, %		Identities for Amino Acid Sequences, %	
	With *R. rhodochrous* Strains	With *R. pyridinivorans* Strains	With *R. rhodochrous* Strains	With *R. pyridinivorans* Strains
1	88.72–99.36 (5 *)	89.63–92.09 (10)	92.88–100.00 (15)	95.17–99.49 (13)
2	95.75–99.92 (6)	95.44–97.91 (15)	97.67–100.00 (5)	97.21–99.07 (7)
3	95.84–99.85 (6)	95.92 (1)	98.50–100.00 (6)	98.44 (1)
4	93.19–100.00 (3)	82.05–93.19 (4)	56.36–100.00 (12)	56.11–96.70 (27)
5	96.16–97.10 (5)	95.14–96.74 (15)	97.17–100.00 (10)	96.95–98.26 (17)
6	Not found	99.85–99.92 (4)	94.55 (1)	99.77 (1)
7	95.43–96.70 (6)	95.73–96.55 (15)	61.56–99.55 (9)	97.52–98.37 (9)
8	97.11–99.67 (6)	96.29–98.76 (15)	85.11–99.26 (6)	95.45–99.75 (5)
9	99.92 (1)	99.35 (1)	100.00 (1)	98.78 (1)

A search for similar DNA and protein sequences was performed against standard NCBI databases of nucleotide collection and non-redundant protein sequences using the megablast and blastp programs, respectively. Only the percents of identity at query coverages ≥ 91% are presented. The gene numbers are the same as those in Table 4. * The numbers of the strains found are shown in brackets.

**Table 6:** Genetic surroundings of genes encoding CYP450 enzymes in *R*. *rhodochrous* IEGM 1362.

Gene No.	Nearby Genes			
	Upstream Transcriptional Regulators	Proteins Participating in Electron Transfers and Redox Reactions	Mobile Elements and Transposases	Other
1	IclR family	No	No	Two-component system, L-proline/glycine betaine transporter ProP; enoyl-CoA hydratase; predicted hydrolase/acyltransferase
2	MarR family	CYP450 gene no. 7; oxidoreductase, short-chain dehydrogenase/reductase family	No	Two uncharacterized MFS-type transporters
3	IclR family	TTP-dependent protein, related to E1 component of pyruvate/2-oxoglutarate/acetoin dehydrogenase; thioredoxin reductase; 2 acyl-CoA-dehydrogenases	No	Putative hydrolase; two fluoride ion transporters CrcB; a permease and translocation protein for amino acids; enzymes for synthesis of lipids and fatty acids
4	XRE family	No	One mobile element protein	Putative peptidase; putative transmembrane protein
5	AcrR family	4-Hydroxyphenylpyruvate dioxygenase; alcohol dehydrogenase; 2 acyl-CoA-dehydrogenases; putative short-chain dehydrogenase	No	Other transcriptional regulators; two methylated-DNA--protein-cysteine methyltransferases; RNA polymerase sigma factor RpoE; DNA-3-methyladenine glycosylase II; DNA polymerase I
6	No	Ferredoxin, 2Fe-2S; ferredoxin reductase; alcohol dehydrogenase; succinate-semialdehyde dehydrogenase [NAD] [NADP+]	One mobile element protein	Enzymes for synthesis of lipids and fatty acids
7	AcrR family	Oxidoreductase, short-chain dehydrogenase/reductase family; CYP450 gene no. 1; putative dioxygenase	No	Two peptidases; two-component system; long-chain-fatty-acid-CoA ligase; putative esterase
8	AraC family	1,2-Dihydroxycyclohexa-3,5-diene-1- carboxylate dehydrogenase; oxidoreductase FAD-binding domain protein; benzoate 1,2-dioxygenase alpha and beta subunits; 2-polyprenylphenol hydroxylase; 4-hydroxyphenylacetate 3-monooxygenase; NADH-FMN oxidoreductase; catechol 1,2-dioxygenase	No	Two benzoate transporters; other transcriptional regulators; muconate cycloisomerase; muconolactone isomerase
9	TetR family	Three oxidoreductases, short-chain dehydrogenase/reductase family; 3-oxosteroid 1-dehydrogenase	One mobile element protein	Isochorismatase; enoyl-CoA hydratase; conserved protein associated with acetyl-CoA C-acyltransferase

The gene numbers are the same as those in Table 4.

## Data Availability

The data presented in this study are available on request from the corresponding author. The draft genome sequence data are available at the NCBI under accession number GenBank NZ_JANFQM000000000.1.

## Supplementary Materials

The following supporting information can be downloaded at: https://www.mdpi.com/article/10.3390/genes15080992/s1, Figure S1: Scheme of the terpenoid backbone biosynthesis; Figure S2: A Blast tree for CYP450 amino acid sequences extracted from the genome of *R. rhodochrous* IEGM 1362.
