# Supplementary material for: Bioinformatics Analysis of the Genome of Rhodococcus rhodochrous IEGM 1362, an (−)-Isopulegol Biotransformer
Source: Genes (Basel). 2024 Jul 28;15(8):992. doi: 10.3390/genes15080992 (PMC11354180; doi:10.3390/genes15080992)
Supplement: Supplementary file 1 [file genes-15-00992-s001.zip › genes-3115288-supplementary.pdf]

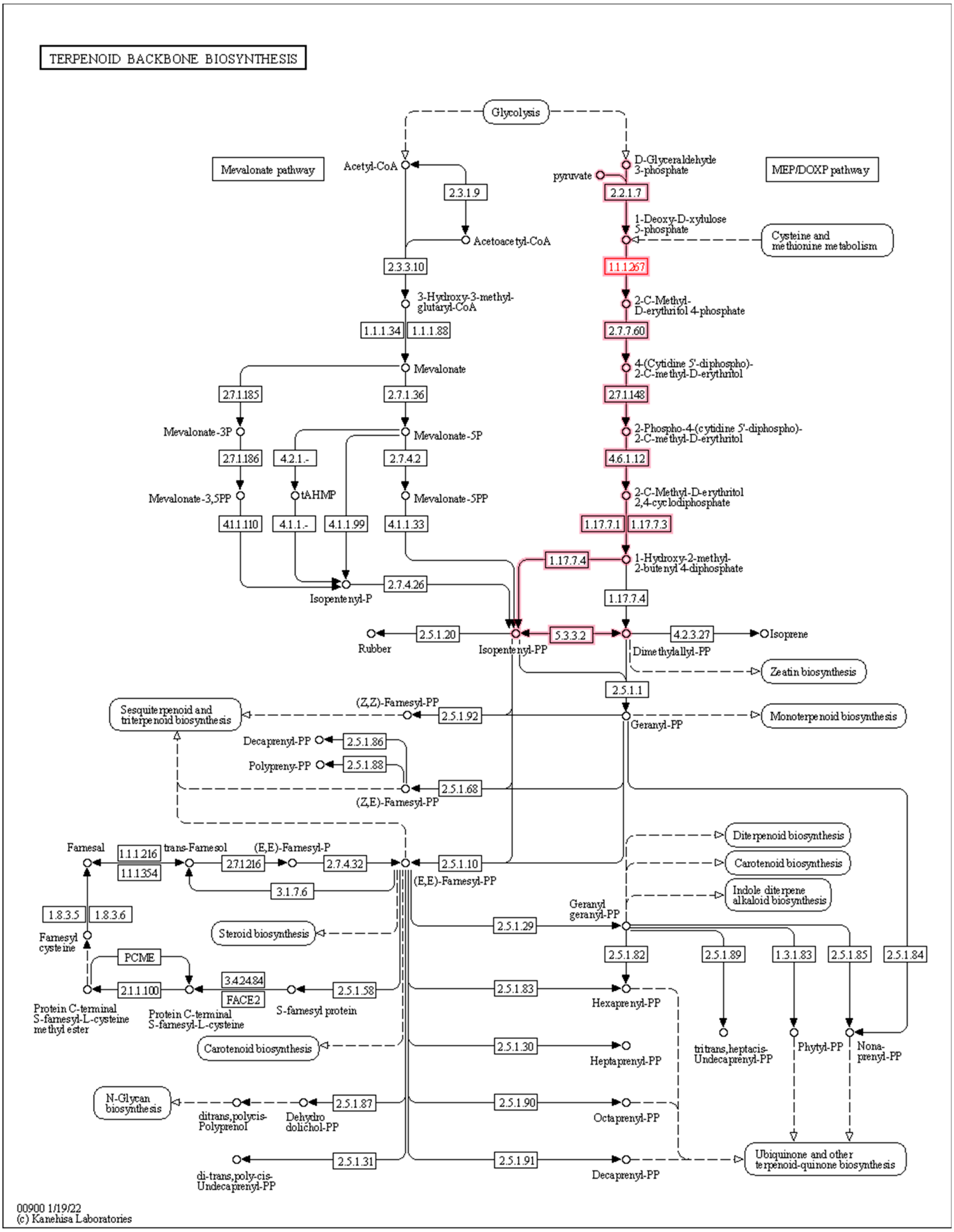


**Figure S1**. Scheme of the terpenoid backbone biosynthesis. The bacterial non-mevalonate pathway for isoprenoid biosynthesis is indicated in pink (https://www.genome.jp/pathway/rn00900)

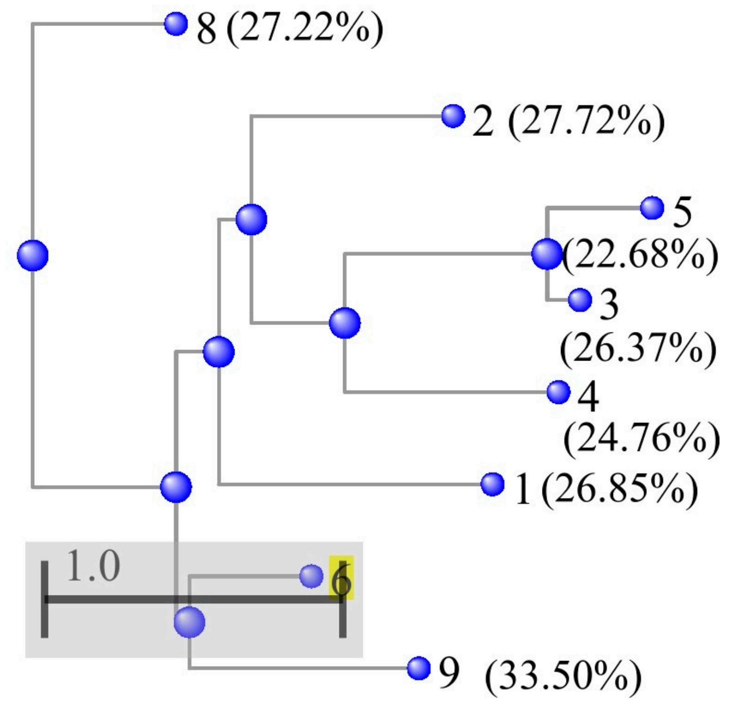


**Figure S2**. A Blast tree for CYP450 amino acid sequences extracted from the genome of *R. rhodochrous* IEGM 1362. The tree was built using the BLAST pairwise alignments of CYP450 gene no. 6 with CYP450 genes. Tree method was Fast Minimum Evolution, distance - Grishin (protein). Percents of identity are shown in brackets.
